# Paediatric and adolescent vulvar lichen sclerosus: delay in diagnosis

**DOI:** 10.1007/s00431-025-06063-2

**Published:** 2025-03-08

**Authors:** Victoria L. Crofts, Dehlia Moussaoui, Viktoriia Shynkarova, Michal Yaron

**Affiliations:** 1 Division of Gynaecology, Department of Woman, Child and Adolescent Medicine, Geneva University Hospitals, Geneva, Switzerland; 2 Division of Paediatrics, Department of Woman, Child and Adolescent Medicine, Geneva University Hospitals, Geneva, Switzerland

**Keywords:** Vulvar Lichen Sclerosus, Paediatric and adolescent gynaecology, Paediatric dermatology, Prepubertal vulvar affection, Pruritis, Vulva

## Abstract

Vulvar Lichen Sclerosus(VLS) is an uncommon, often misdiagnosed condition with a chronic course. Children presenting with VLS may have a wide variety of complaints, which complicates diagnosis. The differentiation of symptoms in the course of VLS causes great diagnostic difficulties. Delayed diagnosis may have an impact on vulvar architecture and long-term sexual health, and can often lead to frustration for both the patient and her parents. The aim of this study was to determine the reasons for delayed diagnosis of VLS in girls and adolescents and to investigate the number of different doctors and specialists consulted before the diagnosis of VLS, the symptoms reported, and the clinical presentation at time of diagnosis. We conducted a retrospective descriptive unicentric cohort study by reviewing medical charts of paediatric and adolescent girls diagnosed with VLS in a tertiary Swiss Centre. The average delay from first symptoms to VLS diagnosis was three years. Many symptoms went unrecognised despite consultations with a wide range of specialists. Once topical corticosteroids were prescribed, relief of symptoms was very quick, usually within one month.

*Conclusion*: Even for a wealthy country such as Switzerland, with ample access to healthcare and specialists, it still takes a long time to arrive at the correct diagnosis and treatment of VLS. Improving knowledge and understanding of VLS disease among paediatricians and healthcare providers could lead to earlier diagnosis and treatment, and thereby significantly improve patient outcomes.

**Table:** 

**What is Known:** • *VLS is a chronic, often misdiagnosed condition with a variety of symptoms in children.* • *Delayed diagnosis can impact vulvar anatomy and long-term sexual health.*
**What is New:** • *In Switzerland (Canton od Geneva), the average delay from symptoms to diagnosis is three years, despite specialist access.* • *Many patients see multiple doctors before diagnosis, highlighting the need for better awareness among healthcare providers.*

**What is Known:**

•  *VLS is a chronic, often misdiagnosed condition with a variety of symptoms in children.*

•  *Delayed diagnosis can impact vulvar anatomy and long-term sexual health.*

**What is New:**

•  *In Switzerland (Canton od Geneva), the average delay from symptoms to diagnosis is three years, despite specialist access.*

• *Many patients see multiple doctors before diagnosis, highlighting the need for better awareness among healthcare providers.*

## Introduction

Vulvar Lichen Sclerosus (VLS) is a chronic dermatologic vulvar condition. The aetiopathogenesis of VLS remains unknown and is probably multifactorial. Associations have been identified between VLS, autoimmune diseases and genetic factors [1–4].

The exact prevalence of VLS is unknown and difficult to determine, as many patients are asymptomatic or misdiagnosed, but it has been reported to be around 1 in 900 [5, 6].

Approximately 10%−15% of all VLS cases present in girls prior to puberty [7]. The average age of VLS onset in girls is 4 to 7 years. Children presenting with VLS may have a wide variety of complaints, among which are vulvar irritation and pain, vulvar pruritus, bleeding due to skin fissures, dysuria, painful defecation, and constipation [8–10].

Some of the symptoms can spontaneously recede after menarche, and the course of the disease can be latent [4, 11]. Nevertheless, the complete disappearance of VLS lesions is rare, and relapses are common [8].

In prepubertal girls with VLS, a three months treatment with ultra potent topic corticosteroids is recommended, followed by a long-term steroid maintenance regimen. This is more aggressive than the guidelines for adult women but seems to offer a chance of complete symptomatic and clinical resolution [12]. Long-term follow up is recommended at least until puberty to ensure that patients are given the best chance of avoiding anatomical abnormalities [13].

Since VLS symptoms are often nonspecific, the diagnosis of VLS can be both challenging and delayed. This delayed diagnosis is not without consequences as it a) increases the risk of long-term vulval architectural changes, b) may decrease quality of life and sexual function, and c) increases the long-term risk of developing squamous cell carcinoma (SCC). These risks, exacerbated by considerable psychological burden [14], highlight the need for early recognition and timely intervention to improve patients’ outcomes.

This retrospective chart review aims to determine the reasons for the delayed diagnosis of VLS in girls and adolescents by investigating a) the range of doctors and specialists consulted before the diagnosis of VLS, b) the symptoms reported, and c) the clinical presentation at the time of diagnosis The insights gained should help to improve knowledge and awareness among healthcare practitioners. This, in turn, may lead to earlier diagnosis, more timely treatment, and better support for patients, ultimately reducing the burden of this condition.

## Method

We performed a retrospective cohort study in a tertiary Swiss centre that offers paediatric and adolescent gynaecology consultation, and paediatric dermatology consultation, caring for children and adolescents up to the age of 18 years.

Inclusion criteria were girls and adolescents who had a clinical or histopathological (confirmed on biopsy) diagnosis of VLS before the age of 18 and had at least one consultation for VLS by a paediatric gynaecologist or paediatric dermatologist at the centre between 2007 and 2023. Clinical diagnosis was based on the typical criteria: external gynaecological examination, including ivory-white skin, genital erosion, purpura, hyperkeratosis, lichenification, atrophy, and disturbance of the vulvar architecture. Follow-up records were collected up to 31.12.2023, the date of the patient’s discharge or failure to follow-up, whichever occurred earliest.

An electronic search for keywords including “lichen sclerosus” and “LS”, was performed in the electronic medical records with a time and age filter (2007–2023 and < 18 years respectively) to identify patients. Medical records were then manually reviewed to confirm eligibility according to our inclusion criteria.

The primary outcome was the delay from the onset of symptoms to the diagnosis of VLS. The date of the initial onset of symptoms was reported by patients to the centre specialist and documented in their medical records. The date of VLS diagnosis was recorded as the first consultation where the condition was explicitly diagnosed.

Our secondary objectives were to determine the range of doctors and specialists consulted before diagnosis of VLS. Additionally, we examined the symptoms reported and the clinical presentation at the time of diagnosis.

The following variables were extracted from medical files: Demographic characteristics; Comorbidities and past medical history; History of lichen sclerosus including age of first symptoms, other health practitioner consulted prior to paediatric gynaecology or dermatology referral in our centre, types of different diagnoses considered prior to VLS; Clinical presentation with initial symptoms, clinical appearance; Treatment: induction, maintenance treatment and response, compliance.

The study was approved by the local Ethics Committee (Project-ID 2021–02365).

We report categorical variables as a number and percentage. Due to non-normal distribution (checked graphically), we report continuous variables as median and interquartile range. We performed all analyses using R Statistical Software (version 4.3.1; R Core Team, 2021).

## Results

### Patient characteristics

A total of 44 patients diagnosed with VLS were included in the study. Among them, 68.3% (*n* = 28/44) were of Caucasian ethnicity, while 14.6% (*n* = 6/44) were Asian, 9.8% (*n* = 4/44) were of African descent, and 7.3% (*n* = 3/44) were classified as other ethnicities.

Only one patient was found to have a concomitant autoimmune condition, specifically celiac disease, while 13.6% (n = 6/44) had other dermatological conditions such as atopic dermatitis and eczema. In addition, 27.3% of the patients (*n* = 12/44) reported a family history of dermatological conditions, which included VLS, psoriasis, eczema and vitiligo. Furthermore, 15.9% of the patients (*n* = 7/44) also reported a family history of autoimmune conditions which included celiac disease, hypothyroidism, allergic asthma and rheumatoid arthritis.

### Disease onset and diagnosis

Most patients, 90.9% (n = 40/44), were pre-pubertal at the time of diagnosis, with only 9.1% (n = 4/44) being pubertal. The average delay between the onset of symptoms and the diagnosis of VLS was three years. The median age at which the first symptoms appeared was 4.4 years (IQR 3.8–8.0), and the median age at diagnosis was 7.4 years (IQR 4.9–10.3).

Before reaching VLS definitive diagnosis, patients were seen by a variety of specialists throughout their diagnostic quest, including adult dermatologists, adult gynaecologists, endocrinologists, gastroenterologists, allergists, psychiatrists, general practitioners, and paediatricians. Some families reported having consulted specialists in alternative medicine such as homeopaths. The average number of specialists consulted and the total number of visits prior to diagnosis were not documented in the medical records.

Presenting symptoms, differential diagnoses considered prior to VLS, and clinical features at the time of VLS diagnosis are summarised in Tables 1, 2 and 3. For all our patients, VLS diagnosis was based on clinical observation by a specialised paediatric gynaecologist or a paediatric dermatologist, both working at the same tertiary centre. None required a biopsy.

**Table 1:** Presenting symptoms at the time of VLS diagnosis according to age

	Total (*n* = 44)	≤ 10 years old *n* = 32/44 (72.7%)	> 10 years old *n *= 12/44 (27.3%)
symptoms	n (%)	n (%)	n (%)
Vulvar itching	38 (86.4)	28 (87.5)	10 (83.3)
Skin changes	23 (52.3)	17 (53.1)	6 (50.0)
Dysuria	17 (38.6)	13 (40.6)	4 (33.3)
Genital soreness	16 (36.4)	11 (34.4)	5 (41.7)
Vulvodynia	13 (29.5)	9 (28.1)	4 (33.3)
Constipation	11 (25.0)	10 (31.2)	1 (8.3)
Bleeding	9 (20.5)	9 (28.1)	0
Malodorous discharge	6 (13.6)	4 (12.5)	2 (16.7)
Urinary incontinence	4 (9.1)	3 (9.4)	1 (8.3)

None of the patients were asymptomatic at the time of diagnosis

**Table 2:** Clinical presentation at time of diagnosis (*n *= 44)

Signs	*n*	%
Depigmentation	36	81.8
Fissures	16	36.4
Erythematous plaques	15	34.1
Thickening of the clitoral hood	14	31.8
Erosion	13	29.5
Scarring	12	27.3
Loss of vulvar architecture	9	20.5
Ecchymosis	6	13.6
Destruction or resorption of the labia	6	13.6
Burying of the clitoral hood	4	9.1
Narrowed introitus	4	9.1

**Table 3:** Differential diagnoses (*n* = 44)

Diagnoses	*n*	%
Vulvovaginitis	31	70.5
Vulval mycosis	16	36.4
Pinworms	10	22.7
Urinary tract infections	10	22.7
Other dermatological conditions	6	13.6
Gastrointestinal condition	6	13.6
Sexual assault	5	11.4
Trauma	4	9.1

Diagnoses considered before reaching VLS diagnosis

We were unable to determine the exact symptoms present at onset versus those at the time of diagnosis, as this information was not detailed in the medical files. Additionally, after years of medical uncertainty, patients often reported multiple complaints but struggled to recall which symptom had appeared first. However, in Table 1, we were able to categorise symptoms by age group. Among our cohort, 40 patients were premenarchal and four were post menarchal, leading us to define two age groups: ≤ 10 years (*n* = 32) and > 10 years (*n* = 12). Notably, all patients presenting with bleeding were under 10 years old. Constipation was also more common in this age group, affecting 31% of patients compared to only 8% in those over 10 years old.

### Management

Treatment with corticosteroid cream was initiated for all patients with a regimen of once daily for at least two weeks or until symptom relief. For induction therapy, 54.5% (*n* = 24/44) of patients were treated with Clobetasol Propionate corticosteroids (class four), 40.9% (*n* = 18/44) with Betamethasone valerate or Mometasone furoate corticosteroids (class three), and 4.5% (*n* = 2/44) with Hydrocortisone acetate corticosteroids (class one). Maintenance therapy with application of the cream once or twice per week involved class four corticosteroids in 51.4% of patients (*n* = 19/44), class three in 45.9% (*n* = 17/44), and class one in 2.7% (*n* = 1/44), with 7 cases missing data. A lower class of corticosteroid was chosen when patients could not tolerate the stronger, class four treatment due to its potency. Symptom relief was reported by patients and caregivers in a median time of one month (IQR 1–2) after the initiation of treatment. Patients and their caregivers were instructed to use the cream once symptoms or signs re-appeared and to return for a check-up.

All nine patients who presented with loss of vulvar architecture at the time of diagnosis experienced symptomatic improvement with treatment compliance, although only partial improvement in vulvar architecture was observed. Upon reviewing the medical files, improvement was recorded subjectively, with descriptions including factors such as enhanced vulvar trophicity, increased mobility of the clitoral hood and vulvar ridges, and reduced vulvar depigmentation.

Following diagnosis of VLS, patients were followed for a median duration of three months (IQR 1–7.5). A significant portion of patients (77.3%, *n* = 34/44) were lost to follow-up and one patient was formally discharged from care. Patients were advised to return if their symptoms reappeared. However, systematic follow-up for asymptomatic patients was not implemented.

## Discussion

This study examines the diagnostic challenges in paediatric VLS, highlighting a significant diagnostic delay, frequent medical consultations prior to accurate diagnosis and high rates of patient loss to follow-up.

Our findings indicate an average diagnostic delay of three years for paediatric VLS, which is significantly longer than the one to two years typically cited in the literature [8, 9]. The longer delay observed in our cohort is particularly noteworthy given that this study took place in a high-income country with relatively easy access to healthcare services. Furthermore, in Switzerland, children under 18 years old can freely access a specialist without requiring a referral from their general practitionner. This suggests that despite the availability of healthcare resources, there may be gaps in awareness, recognition, or prioritisation of VLS in paediatric populations, even among frontline providers in well-resourced settings.

This prolonged delay has potential clinical implications. Studies have suggested that earlier treatment of VLS can prevent symptom progression and reduce the likelihood of long-term sequelae, including vulvar scarring and loss of architecture [15]. Unfortunately, the follow-up in our study was insufficient to determine the long-term evolution of VLS in our cohort.

Our data also reveal that patients often consulted multiple specialists, including adult dermatologists, adult gynaecologists, endocrinologists, gastroenterologists, allergists, psychiatrists, general practitioners, paediatricians, and specialists in alternative medicine such as homeopaths, before receiving a definitive diagnosis from either a paediatric gynaecologist or dermatologist. This "medical wandering" aligns with existing literature, where paediatric VLS patients typically see several specialists before diagnosis [16]. It interesting to highlight that even though some patients had been seen by adult gynaecologists and dermatologists, definitive diagnosis of VLS was solely made by the trained specialist in paediatric gynaecology or the paediatric dermatologist.

These findings highlight the need to prioritise educational efforts among general practitioners, paediatricians and other healthcare providers exposed to childhood diseases, to minimise diagnostic delays.

One potential reason for this prolonged diagnostic journey is the broad and sometimes non-specific nature of VLS symptoms, which can mimic other, more common paediatric conditions. Symptoms such as constipation, urinary complaints, and pruritus may initially be interpreted as unrelated to VLS, leading clinicians to consider more prevalent diagnoses, such as urinary tract infections vulvovaginitis or functional constipation [10, 16]. The variability and subtlety of VLS symptoms, especially in premenarchal girls, can make early recognition challenging and contribute to patients being referred to multiple specialties before reaching the appropriate diagnosis.

To address these diagnostic inefficiencies, the SWIFT model has been developed to streamline identification of VLS in cases where patients present with combinations of urinary and gastrointestinal symptoms [17]. This model proposes that paediatric patients with lower urinary tract symptoms and constipation should be evaluated for VLS, and conversely, those diagnosed with VLS should be assessed for possible lower urinary tract symptoms and gastrointestinal complaints.

Our findings support this model, as many patients in our cohort presented with dysuria and constipation. By establishing clinical guidelines that alert practitioners to the association between lower urinary tract symptoms, constipation, and VLS, we may reduce the frequency of consultations with multiple specialties. This in turn could decrease diagnostic delays and improve the timeliness of effective treatment, thereby preventing long term complication such as architectural changes, impact on quality of life, sexual dysfunction, and development of SCC [13, 14, 18].

A 2020 systematic review found only 37 publications addressing the long-term implications of VLS diagnosed in childhood. These publications outline various aspects of the disease, including the risk of malignancy following VLS during childhood, its relation to puberty, the persistence of symptoms, scarring and architectural changes, other complications, the impact of therapy on complaints, and effects on quality of life. Maintenance treatment of VLS with topical corticosteroids of various potencies tailored to the needs of each individual patient may improve the long-term outcome of the disease, as has been shown for adult women [19].

The goal of treatment is to reduce symptoms, achieve normal texture and normal or near-normal colour, prevent or stabilise architectural change, and reduce the risk of SCC [20].

The literature on maintenance therapy in children is limited. Most studies describe using clobetasol propionate intermittently, based on the recurrence of symptoms, rather than continuous treatment. However, there is inconsistency in how these studies report measurable indicators of disease severity, rates of relapse, and the effectiveness of the treatment over the long term [13, 18–21].

Finally, in our study we found that once appropriate treatment was initiated, symptom relief followed quickly, usually within one month. However, we found that there was a high rate of patients lost to follow-up, with 77.3% of cases not returning for continued care. Similar trends have been reported in the literature, where patients with VLS often do not maintain regular follow-up appointments after the initial improvement of symptoms [8, 22]. This raises questions about whether patients are lost due to symptom resolution, or dissatisfaction with the effectiveness of the treatment. Given that VLS is a chronic condition with the potential to persist or progress, understanding and addressing these follow-up patterns is critical [23].

Regular follow-up is particularly important in VLS because of the long-term risk of vulvar carcinoma, estimated at around 4–5% in adulthood, although this risk has not been reported in paediatric cases [8, 22]. For children and their caregivers, clear communication about the chronic nature of VLS and the need for periodic monitoring (at least once or twice per year, even in asymptomatic patients), may help reduce loss to follow-up, progression of the disease and architectural changes that can be detrimental to patients’ future sexual development [19, 20, 22, 23].

Given that physical examination is essential for monitoring VLS, we recommend that follow-up should be managed by trusted healthcare providers who have been educated about VLS and the importance of regular observation. A study of paediatric trainees identified a lack of knowledge, comfort, and confidence relating to prepubertal vulvovaginal conditions, compared to general medical topics [24]. Ensuring that these providers are aware of the potential complications of VLS and the importance of regular monitoring could improve long-term follow-up and continuity of care for these patients. This might also prevent loss to follow-up, ensuring that patients continue to receive appropriate care as they transition through different stages of life.

This study has several limitations, which may impact the accuracy and comprehensiveness of our findings. Firstly, this is a retrospective study and we are limited by the quality and completeness of the data available in patients’ records. Some data was missing or incomplete, and certain details have not been recorded consistently across all cases. This limitation may impact our ability to capture the full scope of symptom presentation, diagnostic details, and treatment outcomes. Prospective studies could provide a more robust dataset, allowing for more accurate conclusions regarding the progression and treatment outcomes in paediatric VLS. Secondly, a large portion of our patients were lost to follow-up, which significantly limits our understanding of the long-term course of VLS in this population. Without adequate follow-up data we are unable to make a comprehensive assessment of the disease’s progression under treatment, monitor for potential relapses, and examine outcomes as patients age beyond paediatric care. This gap in follow-up data restricts our ability to draw conclusions from our cohort about the long-term efficacy of treatments and the risk of recurrence, both of which are critical for informing clinical guidelines and patient management strategies. To address this limitation, we plan to re-contact these patients and encourage them to return for follow-up visits.

## Conclusion

In conclusion, for our in our cohort of prepubertal girls, we found a delay of 3 years between the onset of symptoms and diagnosis of VLS. Improving education about and awareness of VLS disease among general practitioners and healthcare providers could enable earlier diagnosis and more timely treatment. This, in turn, would spare patients years of unnecessary suffering from chronic symptoms, particularly since appropriate treatment provides rapid relief. Improving understanding, among both patients and clinicians, would significantly improve patient outcomes.

Further prospective studies with long-term follow-up are needed for patients diagnosed with VLS before puberty, continuing through puberty and into adulthood, to better understand the natural history and progression of VLS over time.

## Data Availability

No datasets were generated or analysed during the current study.

## Abbreviations

SCC: Squamous cell carcinoma
SWIFT: Soreness (S), whitening (W), urinary incontinence (I), fissures (F), and thickening of the clitoral hood (T)
VLS: Vulvar Lichen Sclerosus
